# A special issue of Essays in Biochemistry on proteasome and protein degradation

**DOI:** 10.1042/EBC20260013

**Published:** 2026-05-14

**Authors:** Indrajit Sahu, Michael H. Glickman

**Affiliations:** 1 National Centre for Cell Science (BRIC-NCCS), Pune, Maharashtra, India; 2 Faculty of Biology, Israel Institute of Technolog—Technion, Haifa, Israel

## Abstract

Research on the ubiquitin–proteasome system (UPS) is expanding exponentially across the subcontinents of Asia, including China, India, Israel, Korea, and Japan, as evidenced by recent conferences held in Asia. In the present special issue, we have compiled several review articles on the recent research developments in the UPS field from the conference speakers in the last two proteasome conferences organized during late 2024 and early 2025 in Asia—the XII-ZOMES conference in Shenzhen, China, and ProUPS-25 in Chennai, India. Together, these reviews emphasize the complex mechanisms of post-translational modifications, ubiquitin signaling, and specialized proteasome functions through which the UPS regulates diverse processes, including proteostasis, gene expression, immunity, plant development, mitochondrial dynamics and function, and disease pathways such as cancer, fibrosis, and neurodegeneration. Finally, the present special issue underscores the growing therapeutic potential of targeting the UPS, including proteasome inhibitors and emerging technologies like PROTACs (proteolysis targeting chimeras), positioning the system as a key platform for developing precision treatments for a wide range of diseases.

## Introduction

The ubiquitin–proteasome system (UPS) has undergone transformative advances since its initial characterization. Early seminal work in the late 1970s to 1980s established ATP-dependent proteolysis distinct from lysosomal pathways, leading to the discovery of ubiquitin as the covalent tag for protein degradation and the 26S proteasome as the central proteolytic machinery [1,2]. Pioneering elucidation of the ubiquitination cascade (E1 activating, E2 conjugating, and E3 ligase enzymes) by Hershko, Ciechanover, and Rose provided a mechanistic framework for selective substrate targeting and earned these pioneers the 2004 Nobel Prize in Chemistry [3]. After the discovery and characterization of ‘thymopoetin’ (ubiquitin) by Gideon Goldstein and the multicatalytic protein complex (proteasome) by Alfred Goldberg during the late 1970s, there has been a surge in research efforts to push the boundaries of understanding this ATP-dependent proteolytic pathway in eukaryotic cells [4,5]. Structural biology milestones, notably high-resolution X-ray crystallography of the 20S core and cryo-EM reconstructions of the full 26S holoenzyme, revealed proteasome architecture, substrate translocation, and regulatory particle dynamics.

The proteasome is the major protein-degradation machine in the UPS in all eukaryotes. Interestingly, the structure and function of the proteasome are remarkably conserved across all eukaryotes, and similar degradation complexes exist in primitive unicellular organisms. In this special edition of Proteasome and Protein Degradation, the invited authors cover several aspects of the UPS, collating several review articles on our understanding of the mechanism of cellular proteolysis.

Dubiel et al. highlight how several variants (CSN^CSN7A^ and CSN^CSN7B^) of COP9 signalosome (CSN) regulate proteostasis by modulating cullin-RING ubiquitin ligases (CRLs). CSN^CSN7A^ and CSN^CSN7B^ selectively associate with CRL3 and CRL4A, respectively, via their C-terminal domains [6]. During adipogenesis, these latent CSN–CRL complexes acquire specialized roles; CSN^CSN7A^–CRL3 is recruited to lipid droplets and neddylated, whereas CSN^CSN7B^–CRL4 dissociates from its substrate receptor to halt p27 degradation and promote cell cycle arrest, after which inactive complexes are ubiquitylated and cleared by selective macroautophagy, a process enhanced upon CSN5 inhibition.

Kim et al. explain how the 26S proteasome plays a central yet mechanistically unresolved role in large-scale proteome remodeling during processes such as fibrosis and cellular differentiation [7]. The immunoproteasome, a specialized variant incorporating alternative catalytic subunits, expands antigen presentation capacity and exhibits distinct substrate specificities via specific ubiquitin linkages or ubiquitin-independent pathways, thereby promoting proteome reprogramming and contributing to the transition toward a simplified fibrotic proteome in affected tissues.

In their review article, Zhang et al. emphasize how nucleic acid modifications (e.g., m6A, m5C, m3C, DNA methylation, deamination, and oxidation) and protein post-translational modifications (including ubiquitination, SUMOylation, acetylation, methylation, and phosphorylation) coordinately and context-dependently regulate R-loop formation and resolution, thereby influencing genome stability, gene expression, and disease pathogenesis [8].

Ravichandran et al. describe the post-translational modification by ubiquitin or the ubiquitin-like modifier FAT10, which not only serves as a targeting-signals but also actively reshapes the thermodynamic and conformational landscapes of substrate proteins in a site-dependent manner [9]. Ubiquitin destabilizes proteins through entropic or enthalpic effects, often requiring unfoldases such as p97/VCP for degradation, whereas FAT10’s intrinsic instability promotes unfoldase-independent degradation, redefining ubiquitin-like tagging as an active regulator of protein fate with therapeutic implications.

Shimron et al. highlight recent evidence identifying p62/Sequestosome-1 as a critical regulator that enhances UPS efficiency by forming dynamic nuclear condensates via liquid–liquid phase separation, which concentrates 26S proteasomes and ubiquitinated substrates to promote efficient degradation [10]. These p62 condensates facilitate turnover of oncogenic and stress-associated proteins, such as enabling c-Myc degradation through recruitment of SCF^Fbxw7^ and stabilizing PML nuclear bodies by targeting RNF4, thereby coordinating nuclear protein quality control and protecting cells from proteotoxic and oncogenic stress.

Zhou et al. elaborate on how the UPS in plants enables the transition between skotomorphogenesis and photomorphogenesis by precisely regulating key light signaling components [11]. In darkness, E3 ligases such as CRL4^COP1–SPA^ target photomorphogenesispromoting factors like HY5 for degradation, whereas under light conditions, E3 ligases, including CRL1^EBF1/2^ mediate the degradation of inhibitory regulators such as phytochromeinteracting factors, thereby ensuring proper photomorphogenic development.

Mitsiadou et al. discuss how protein quality control systems preserve proteostasis under stress by coordinating responses such as stress granule (SG) assembly, whose dysregulation contributes to neurodegenerative diseases, including amyotrophic lateral sclerosis (ALS) [12]. They describe evidence identifying the NEDD8 pathway, particularly the deconjugating enzyme NEDP1, as a critical regulator of SG clearance, where NEDP1 inhibition promotes hyper-NEDDylation of PARP1 to enhance SG turnover and toxic aggregate disassembly without compromising DNA repair. These findings implicate NEDP1 as a promising therapeutic target for ALS in the near future.

In their review, Singh et al. summarize the importance of E3 ubiquitin ligases in cancer cell metastasis [13]. Several E3 ligases act as promoters, while others act as repressors of metastasis. Their article focuses on the potential role of E3 ubiquitin ligases in cancer metastasis and reveals their molecular functions and targets, which are crucial for anti-cancer therapeutic interventions.

Ram et al. highlight the AAA+ ATPase p97/VCP as a central regulator of mitochondrial homeostasis, coordinating mitochondria-associated degradation, mitochondrial dynamics, apoptotic signaling, mitophagy, and ER–mitochondria contact site regulation through adaptor-mediated extraction [14]. Given its involvement in organellar cross-talk, pathogen interactions, and RNA-mediated regulation and its mutation in ALS and frontotemporal dementia, p97 functions as an integrative hub linking mitochondrial proteostasis to cell fate decisions. Evidently, p97 represents a promising therapeutic target in metabolic and neurodegenerative diseases.

Rusnac et al. elaborate that inositol hexakisphosphate (IP6), as a multifunctional regulator that directly modulates components of the UPS [15]. Acting as an allosteric effector, molecular glue, or prosthetic group, IP6 binds specific proteins and multiprotein complexes, thereby controlling their structural and functional dynamics, highlighting a broader and potentially translationally relevant role for soluble inositol polyphosphates in UPS regulation.

Sundararajan et al. focus on PSMD9, a 19S proteasomal chaperon, and discuss its noncanonical role beyond proteasome assembly function, which has emerging functions in cancer-related proteasome dynamics, ubiquitin-mediated degradation, unfolded protein response, proteostasis, and nucleolar organization [16]. Although lacking enzymatic activity, PSMD9 exerts multifunctional effects through extensive protein–protein interactions, with genomic and proteomic studies implicating it in diverse diseases and identifying it as a potential vulnerability in certain solid tumors.

Serawat et al., in their review, provide a comprehensive overview of the models proposed over the past decade to explain Parkin’s autoinhibition and activation [17]. They summarize the key structural and biophysical studies that have progressively uncovered the molecular basis of Parkin activation, tracing the evolution of these insights. The present review concludes by discussing the intriguing and still unresolved question of whether Parkin activation occurs through a* cis*- or* trans*-mechanism and outlines future directions for research aimed at understanding these pathways.

Feifei et al. summarize the recent advances in endosomal sorting complex required for transport (ESCRT)-mediated signaling in plant abiotic stress responses, highlighting the fundamental roles of ESCRT components in plant biology and providing key targets for molecular breeding of abiotic stress-tolerant crops [18].

Sahoo et al. focus in their review on the role of ubiquitin modifications and their associated regulators impacting protein fate and neurotoxicity and highlight the current therapeutic strategies targeting the degradation of aggregating proteins to uncover potential avenues for treating neurodegenerative diseases [19]. The review examines the pivotal role of ubiquitin modifications in altering the fate of aggregation-prone proteins, including tau, α-synuclein, mutant huntingtin, TAR DNA-binding protein 43, and superoxide dismutase.

Gupta et al. review the key developments in our understanding of how specific ubiquitin linkage types participate in various cellular pathways and their implications on the fate and function of the protein [20]. The topologically diverse polyubiquitin chains function as discrete molecular signals, each with distinct physiological outcomes.

Taken together, these reviews underscore the UPS as an evolutionarily conserved, highly regulated system essential for cellular proteostasis and a fertile ground for precision therapeutics.

## Open Access

Open access for this article was enabled by the participation of Technion in an all-inclusive Read & Publish agreement with Portland Press and the Biochemical Society under an agreement with MALMAD.

## Abbreviations

ALS: amyotrophic lateral sclerosis
CSN: COP9 signalosome
ESCRT: endosomal sorting complex required for transport
IP6: inositol hexakisphosphate
UPS: ubiquitin–proteasome system
